# The Secret Lives of Miniature Batteries

**DOI:** 10.3390/s24030748

**Published:** 2024-01-24

**Authors:** Sivan Toledo, Shai Mendel

**Affiliations:** The Blavatnik School of Computer Science, Tel Aviv University, Tel Aviv 69978, Israel

**Keywords:** batteries, internal resistance, reservoir capacitor, current limiting, concentration polarization, wildlife tracking tags, asset tracking tags, implantable, ingestible, injectable devices

## Abstract

This article describes the design, implementation, and use of a new system to investigate the behavior of small batteries that power sensor and wireless systems that consume relatively high power during infrequent short activity periods. The system enables simple, low-cost, long-term (days to weeks) monitoring of batteries under such loads. Data collected by this system revealed a major cause of failures in wildlife tracking tags, an effect called concentration polarization, which causes a transient increase in the internal resistance of the battery. The article describes the goals and the design of the system, failures that it revealed, mechanisms to mitigate the limitations of miniature batteries, as well as a methodology to optimize and validate the design of tags powered by miniature batteries.

## 1. Introduction

Miniature batteries, both primary and rechargeable, remain the dominant power source for implantable, ingestible, and injectable biotelemetry devices [1,2], asset tracking tags [3], wildlife tracking tags [4,5,6], and miniature electronic devices in many other applications. Miniature batteries are far from ideal voltage sources. To be used effectively, their characteristics must be well understood and their limitations should be mitigated using both hardware and firmware mechanisms.

Many types of batteries can supply energy at a limited rate, due to their physical structure and the rate of chemical reactions in the battery. In general, the smaller the battery, the smaller the rate at which it can supply energy. For example, 0.6 g Renata Lithium–Manganese coin-cell CR1025 batteries are rated to source only a 0.05 mA current continuously and a 0.4 mA current at maximum [7], whereas the 8.3 g CR2477N battery from the same series is rated at 1.0 mA and 2.5 mA, respectively [8]. The limited power-delivery capability of small batteries is usually modeled as internal resistance, a resistor in series with an ideal voltage source.

Therefore, miniature batteries are poorly matched to loads that consume high power in short bursts, even if these bursts are infrequent. We refer to such bursts of high power consumption as the load’s activity period or as pulses of activity. Many sensing and tracking devices, especially wireless ones, belong to this category: they consume very little power continuously, but consume significant power for short bursts that are infrequent and often periodic.

This limitation is well known and is usually addressed in pulsed loads by adding a *reservoir capacitor* in parallel with the load [4,6,9,10]. During a high-power activity period, the energy that the load needs is drawn mostly from the capacitor, not the battery. During the following low-power period, the capacitor is recharged from the battery, enabling the next pulse of activity and so on.

Another limitation of small batteries is not well known and has largely been ignored in the sensor-systems literature. Batteries can experience a phenomenon called *concentration polarization*, in which internal resistance increases temporarily because reactants become depleted near the battery’s electrodes [11,12]. This is resolved via diffusion of the reactants, which takes time, often seconds, and sometimes minutes. Concentration polarization is exacerbated by low-impedance loads (attempts to draw a high current), so a reservoir capacitor can increase the likelihood of these transient failures.

A third aspect of small batteries that is also often ignored in the sensor-systems literature is that their behavior is statistical, not deterministic. The statistical behavior is caused by the statistical nature of diffusion and chemical reactions. Testing a few units without observing failures does not imply that the chosen battery can reliably power the load. More extensive testing is required. For example, investigations of a few Zinc–Air batteries by one of the authors [10] led to the conclusion that they can effectively power wildlife tracking tags. However, larger-scale field testing resulted in high failure rates.

This paper proposes a methodology to reveal the intricacies of miniature batteries that power pulsed load, of which it uses this methodology to demonstrate complex battery behaviors and uses these observations to propose and evaluate mitigation strategies. More specifically, the main contributions of this article are

- An inexpensive open-source open-hardware and easy-to-replicate instrument for characterizing the behavior of miniature batteries under pulsed loads, both artificial (with the instrument itself presenting a programmable load) and real. The design allows large-scale battery testing, which is required to fully characterize their statistical behavior.
- The results of extensive testing of miniature batteries, mostly under artificial loads (but not only), to demonstrate the utility of reservoir capacitors and of resistors that limit their charging rates, demonstrate concentration–polarization events, and measure the time it takes to recover from them.
- A circuit-simulation model and a partial analytical model that enable optimization and validation of the choice of reservoir capacitors and limiting resistors given the characteristics of the load and the battery.

### Related Work

Miniature primary batteries are used in a variety of wildlife tracking tags. Some articles on tracking tags discuss the challenging characteristics of such tags and how to adapt to them [4,5,6,9].

Our project builds upon the work of [10], in which the author presented a simpler battery characterization system along with a partial characterization of miniature batteries under pulsed loads. The new system that we present in this article is more accurate and much easier to replicate. It also revealed an important phenomenon, concentration polarization, that the earlier research did not reveal.

Zermout et al. [13] propose a system quite similar to the previous one for testing single rechargeable cells. Their circuit allows for charging the battery, not only discharging it, and it provides battery-temperature feedback to the microcontroller. This is required for testing fast charging and discharging. Our application does not need either feature, but in general, they are certainly useful. For much higher levels of power (up to 48 V and 10 A), Santoro et al. propose a low-cost DC load with a switching topology [14].

At the other extreme, authors use computer-controlled high-end laboratory equipment to characterize batteries [15,16].

## 2. Materials and Methods

This section presents our battery-characterization system and our design aids, which consist of a simulation model along with an analytical model (formulas) for some of the quantities.

To make both the discussion and the examples concrete, we focus on the testing and analysis of batteries that power wildlife tracking tags with integrated RF microcontrollers [6,9], such as the CC1310 from Texas Instruments [17]. We use load parameters taken from actual tracking tags that were deployed in large numbers. Specifically, we assume that the tag transmits a 10 mW signal for 8 ms every few seconds (0.5 s to 16 s). This RF microcontroller and many similar ones require a voltage of 1.8 to 3.8 V, allowing Lithium–Manganese batteries to power them directly, as well as several other types of batteries (e.g., pairs of silver–oxide cells). The microcontroller uses either a linear regulator or a switching step-down regulator to regulate the supply voltage down to about 1.7 V. The CC1310’s data sheet specifies the current consumption while transmitting a 10 mW signal using a 3.6 V power supply and the switching regulator as 13.4 mA. This implies that with the switching regulator, the chip consumes 13.4×3.6=48.24 mW while transmitting. If the linear regulator is used, it will consume approximately 28.38 mA to produce the same power at 1.7 V. We assume that the load requires at least 1.8 V to operate correctly, which is the case for the CC1310. We also assume that the no-load battery voltage is 3 V, typical of Lithium–Manganese batteries.

### 2.1. Battery-Behavior Characterization System

We designed and built a system to characterize the behavior of miniature batteries under a real or simulated load that is typical for wildlife tracking tags. The system consists of hardware (an analog circuit interfaced to a microcontroller), firmware, and host software that drives the testing and collects the results. The system works either in *simulation mode* or in *real-load mode*. The system is an enhancement of an earlier and more limited load simulation system [10]. The software and hardware for this system are open and freely available [18].

#### 2.1.1. Microcontroller Platform

**Figure 1:**
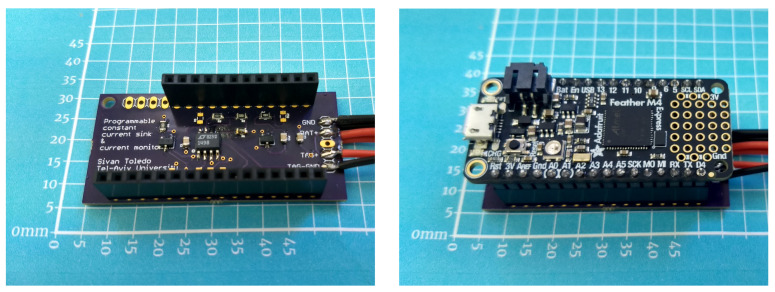
The battery characterization hardware. The image on the left shows the add-on circuit that we designed and built. The image on the right shows the same unit, but stacked below an Adafruit Feather M4 Express microcontroller board.

The hardware consists of a stack of two circuit boards, shown in Figure 1. The bottom one contains a custom analog circuit that we designed, and the top one is a commercial microcontroller board, the *Adafruit Feather M4 Express* [19]. The Adafruit Feather series offers small form-factor boards (24 by 51 mm) with several different microcontrollers, some with WiFi or other radios, and with extension connectors that have the same or almost the same layout in all the boards. Therefore, extension boards like the one that carries our analog circuit can be compatible with multiple Feather boards; in particular, our board is compatible with at least the Express M4 board and with the WICED WiFi board, costing $23 and $35, respectively. The M4 Express board uses a 120 MHz Cortex M4 microcontroller (ATSAMD51J19) with 512 KB Flash and 192 KB RAM.

These Feather boards support the Arduino firmware environment, which is an easy-to-use C++ programming environment that supports numerous microcontroller boards (see, e.g., [20]). The Express M4 board also supports CircuitPython, a more high-level firmware environment. We used the Arduino environment, due to its performance (it produces native C/C++ binaries) because it allows our firmware to run on many other microcontroller boards and for others to easily modify and enhance our code.

The small form factor of the Feather boards makes the hardware unit compact and reduces the cost of the analog circuit board. The size still allows for easy manual assembly of the custom analog board (one of the authors manually soldered 11 of these boards in a few hours).

#### 2.1.2. Circuit Design

**Figure 2:**
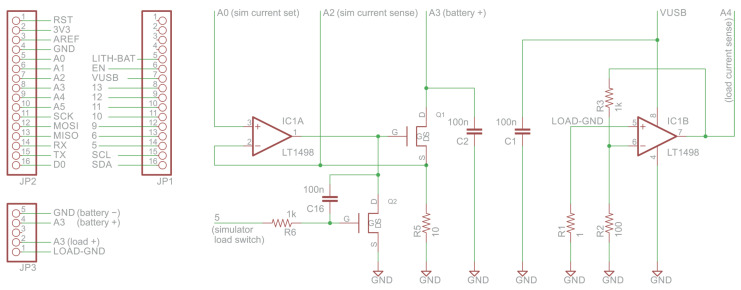
The analog part of the load simulator and battery tester.

The custom analog section of our load tester and battery simulator is shown in Figure 2. Connectors JP1 and JP2 connect the circuit to a microcontroller board from the Adafruit Feather series. Connector JP3 is used to connect the battery under test and, in real-load mode, a load. In simulation mode, analog Pin A0 is used to set the current that the simulator draws from the battery in each pulse; it should be driven by a digital-to-analog converter (DAC). Digital pin 5 controls the current sink: when it is low, the simulator draws the current from the battery; when it is high, the simulator draws no current (but a real load might, if one is connected). Pins A2, A3, and A4 should be connected to analog-to-digital converter (ADC) inputs. Pin A2 monitors the actual current drawn by the simulator, pin A3 monitors the battery voltage (in both simulation and real-load modes), and Pin A4 monitors the current drawn by a real load.

Operational amplifier IC1A together with N-channel MOSFET Q1 and resistor R5 form a programmable current sink. When N-channel MOSFET Q2 conducts, it pulls the gate of Q1 to ground, disconnecting the load resistor R5 from the battery. When pin 5 is low, the negative feedback loop keeps the voltage on R5 at the same level as the voltage that the microcontroller’s DAC places on A0. A voltage of *v* Volts causes R5 to sink v/10 Amperes.

The role of capacitor C16 is to amplify the Miller effect of MOSFET Q2, in order to slow down the turn-on of the current sink. Without it, the current drawn by the load starts with a high short (on the order of a microsecond) spike that settles to the programmable level. This spike, which does not occur under actual loads, can potentially stress the battery. The spike is part of the settling of the operational amplifier, in which the negative feedback loop is open before the pulse (the gate of Q1 is grounded).

A simulation of the circuit indicated that without this Miller capacitor, a 35 mA current-sinking pulse starts with a 270 mA spike lasting 1.5 μs. With the 0.1 μF capacitor, the spike is reduced to 40 mA. Oscilloscope measurements verified these findings. The details of the spike depend on the parameters of both the MOSFET and the amplifier; the simulation used models of the actual parts that we use in the actual circuit, DMN1019 and LT1498.

The circuit around IC1B is simply an 11× amplifier that amplifies the voltage developed across the current-sensing resistor R1. The voltage drop across this resistor causes tags to fail while battery voltage is still above the tag’s threshold (around 1.8 V), but only slightly so, about 20 mV above the threshold.

#### 2.1.3. Firmware, Software, and Protocol Design

In simulation mode, the experiment is driven by a host computer running C-sharp (version 7) software that drives the experiment and collects measurement data. In real-load mode, the system monitors the current consumption and battery voltage of an actual tag. In this mode, the load itself controls the power-consumption scheduling, usually using a microcontroller; our system monitors the current consumption and battery voltage at a high sampling rate and transfers a summary of the measurements to the host computer for logging.

In simulation mode, the user configures the host-based software for a specific pulse-repetition rate, pulse duration, and current to be drawn in each pulse. Each current-sinking pulse is initiated by the host. This ensures that if the host software stops or the host itself halts or reboots, the experiment is suspended and the battery is not drained further until the experiment is restarted. This is important since some of these experiments, especially with large batteries, can last days or weeks. In this mode, the firmware waits for a command from the host to start a pulse. During this wait, it monitors battery voltage and records the highest voltage it has seen. When a command to start the sinking current arrives, the firmware sets the DAC output to the correct level, starts the pulse by clearing pin 5, and samples the battery voltage and the current drawn. The samples are time stamped. At the end of the pulse, the firmware sends to the host the highest voltage before the pulse (and resets this variable), the lowest voltage and highest current during the pulse, and all the samples of the current and voltage during the pulse. The firmware samples the ADC at about 12.5 ks/s (this is a limitation of the Arduino library that drive the ADC; the microcontroller can sample at up to 1000 ks/s), giving about 50 current and voltage samples in each 8 ms pulse.

In this mode, the host software stores all the data in a file and can display both the overall graphs of extreme values in each pulse (highest voltage before the pulse, lowest voltage and highest current during the pulse), as well as the detailed measurements during a pulse that the user selects on the screen. The visualization software is written in Python.

In real-load mode, the firmware discovers the timing of current-drawing pulses automatically using a set of rules. The rule that indicates the beginning of a pulse requires 4 consecutive current samples to exceed a threshold of about 5.4 mA; the rule for terminating a pulse requires 4 samples below 4.5 mA, to provide hysteresis. These thresholds are constrained by the operational amplifier’s errors (mainly offset voltage), which are fairly high for the LT1498. A precision amplifier should allow for using much lower thresholds. At the end of each pulse, the firmware sends the same data as in the simulation mode: extreme values before and during the pulse, and high-resolution measurements during and just before the pulse.

The real-load mode also implements facilities to record the current and voltage behaviors when a load behaves abnormally, which is usually caused by some failure or fault. In normal behavior, typical loads consume the current in short pulses that last up to 30 ms or so. When a load experiences a failure, it can consume the current continuously, which can drain the battery. When a pulse lasts longer than a threshold (currently 30 ms), the firmware moves to slow-sampling mode, in which the system reports every millisecond the maximal current and minimal battery voltage observed in the previous millisecond. The host software records these values but can aggregate them if the failure lasts longer than a few seconds.

### 2.2. Reservoir Capacitor and Current Limiting Design Procedure

Miniature batteries may be unable to source enough current to power a bursty load. Therefore, energy must be transferred from the battery to an intermediate low-impedance energy storage device, typically a reservoir capacitor. As energy is drawn from the capacitor during an activity pulse, the voltage across it drops. If the capacitor is connected in parallel with the battery, it now presents a low-impedance load, risking concentration polarization in the battery. To avoid this, the reservoir capacitor is typically connected to the battery via a current limiter, usually a simple resistor.

The selection of the capacitor and the limiter should start with a characterization of the load: the length of activity periods, their frequency, the minimum supply voltage required by the load, and the current or power consumption of the load. In most cases, the actual load requires a voltage-regulated supply. When the regulation is linear, we model the load as a constant-current sink. With a switching regulator, we model the load as a constant-power sink. It is best to characterize the load using both data-sheet information and actual experiments with a low-impedance power source (e.g., a lab power supply). In the experiments, the current drawn can be evaluated by our system, or by a combination of a stand-alone current-sensing resistor, an amplifier, and an oscilloscope. The use of both data-sheet information and measurements reduced the chances of errors. Finally, the characterization should also include the voltage of the battery at rest (when little or no current is drawn from it).

**Figure 3:**
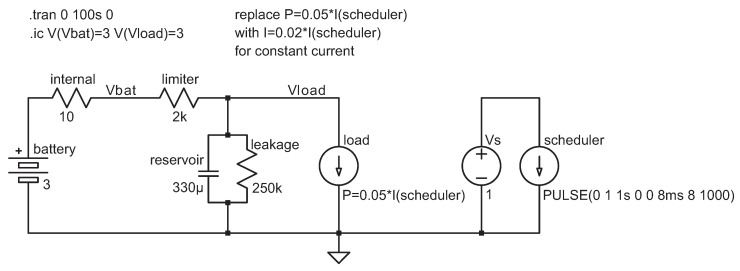
Using a resistor to limit the current sourced from a battery when the load is connected in parallel with a large reservoir capacitor. In this schematic, the load is modeled by a current source. The schematic was captured from an LTSpice simulation model. The asterisk symbol in the formulas represents multiplication.

Figure 3 shows a model of this topology. A bursty load is connected in parallel with a reservoir capacitor. The load is powered by a battery: here, it is a 3 V battery with a 10Ω internal resistance. The load and the capacitor are connected to the battery through a current-limiting resistor: here, it is 2kΩ. Physically small large-value capacitors are leaky. We model the leakage as a 250kΩ resistor (This value is based on a particular tantalum capacitor, the 330 μF, 6.3V Kyocera F950J337KBAAQ2, whose rate leakage is specified as 20.8 μA at the rated voltage at $20^{\circ }$, dropping quickly at lower applied voltages and rising with temperature. The 250 k resistor corresponds to about 12μA, with the leakage at 3 V and $50^{\circ }$, respectively). Figure 3 models the load as a 50 mW constant-power sink during activity periods. This is about right for a CC1310 RF microcontroller transmitting a 10 mW and using its built-in switching regulator. We can easily switch the load to a constant-current source by replacing the load formula in the model. The model also includes a separate voltage source connected to a periodic pulsed current sink, sinking 1 A for 8 ms every 8 s. This periodic current sink is used to modulate the actual load in simulations.

We also considered limiting the battery current using an integrated current regulator (e.g., 1N5283) or a transistor (JFET, MOSFET, or bipolar) configured as a current-limiting regulator. An ideal current regulator might have an advantage over a current-limiting resistor because as the capacitor recharges, the impedance of the regulator drops, reducing the amount of energy wasted in it. However, true regulation only starts when the voltage across a regulator like the 1N5283 reaches a threshold, around 1 V; below that, the regulator does not conduct or it behaves like a resistor. In our case, the regulator rarely sees a voltage higher than 1 V, so it is not likely to have any advantage. Therefore, this does not appear to be a practical solution.

The rest of this section explains how to size both the reservoir capacitor and the current limiting resistor.

#### 2.2.1. Simulation-Based Design

**Figure 4:**
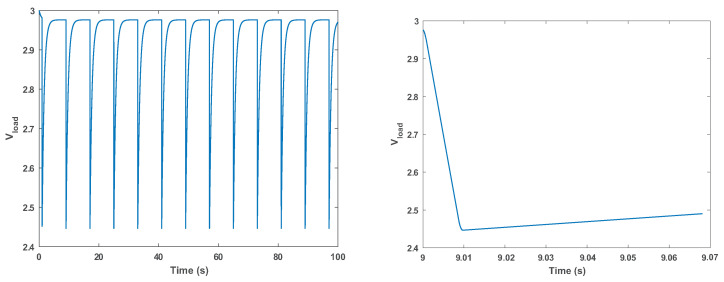
A 100 s simulation of the circuit shown in Figure 3, in which the load sank 20 mA (constant current) for 8 ms every 8 s. On the right, we see the entire 100 s, showing that between current-sinking pulses, the voltage on the capacitor reaches an asymptote close to 3 V. On the right, we see the voltage during and after one pulse. During the pulse, it drops almost linearly due to the Q=CV behavior of the capacitor and the almost linear decrease in charge *Q*. After the pulse, the voltage starts to rise as the capacitor is discharged through the current-limiting resistor.

We advocate choosing the current-limiting resistor and the reservoir capacitor using simulations of the model shown in Figure 3. The file containing the model is freely available [18]. The simulation is carried out using the free LTSpice software version 17.0.30 and takes less than a second. After the simulation, the user can visualize waveforms generated by the simulation, like the voltage drop across the load, shown in Figure 4.

The resistor–capacitor combination is feasible for the given load if the minimum voltage in the simulation stays above the minimum voltage required by the load, plus a reasonable safety margin. In Figure 4, the voltage drops to about 2.45 V but not below; this is well above the 1.8 V minimum of the CC1310, so the combination is feasible. Reducing the capacitor to 100μF causes the voltage to drop below 0.6 V, so this value is certainly not feasible. Longer activity periods and heavier loads require larger capacitors. Increasing the current-limiting resistor slows down the charging of the capacitor and can also lead to the voltage dropping below the threshold.

Our LTSpice model also evaluates several quantities associated with the simulation. The most important one is the minimum voltage across the load, indicating whether the simulated values are feasible. Another is the maximum current drawn from the battery, which indicates the effectiveness of the current limitation. With the values shown in Figure 3, the maximum is less than 253 μA.

Other quantities that the model computes include

(1) $$\begin{array}{ccc} E_{\mathrm{bat}} & = & \int \left|I_{\mathrm{bat}}\right|V_{\mathrm{bat}} \end{array}$$

(2) $$\begin{array}{ccc} \;\;E_{\mathrm{load}} & = & \int \left|I_{\mathrm{load}}\right|V_{\mathrm{load}} \end{array}$$

(3) $$\begin{array}{cccc} \; & E_{\mathrm{limiter}} & = & \int \left|I_{\mathrm{limiter}}\right|\left(V_{\mathrm{bat}}-V_{\mathrm{load}}\right) \end{array}$$

(4) $$\begin{array}{ccc} \;\;\;E_{\mathrm{leakage}} & = & \int \left|I_{\mathrm{leakage}}\right|V_{\mathrm{load}} \end{array}$$

where *E* represents energy, *I* current, *V* voltage, and the integrals are over time. The model also computes the ratio of the last three to the first. These ratios indicate how much energy was transferred from the battery to the load and how much was wasted in the limiter or due to leakage. With the values in Figure 3, these ratios are 54% (only a little over half the energy was transferred to the load), 6.5%, and 33%.

#### 2.2.2. Sizing the Reservoir Capacitor Analytically

The required capacitance can also be analyzed analytically, at least to obtain a good starting value for the simulation. In constant-power mode, the 48.24 mW power consumption translates into a 0.386 mJ energy consumption over the 8 ms activity period. The energy in a capacitor is $E=\left(1/2\right)CV^{2}$, so the voltage $V_{\mathrm{end}}$ at the end of the pulse satisfies the equation

(5) $$\frac{1}{2}CV_{\mathrm{start}}^{2}-0.386\times 10^{-3}=\frac{1}{2}CV_{\mathrm{end}}^{2}\;,$$

where $V_{\mathrm{start}}$ is the voltage on the capacitor at the beginning of the pulse (hopefully the voltage of the battery when it is not under load). If we assume that $V_{\mathrm{start}}=3$ and we constrain $V_{\mathrm{end}}\geq 2.4$, for example, we obtain

(6) $$\begin{array}{ccc} V_{\mathrm{end}}^{2} & = & V_{\mathrm{start}}^{2}-\frac{2\times 0.386\times 10^{-3}}{C}\\ & = & 9-\frac{2\times 0.386\times 10^{-3}}{C}\\ & \geq & 2.4^{2} \end{array}$$

or

(7) $$\begin{array}{ccc} 2.4^{2} & = & 5.76\\ & \leq & 9-\frac{2\times 0.386\times 10^{-3}}{C} \end{array}$$

so

(8) $$\begin{array}{ccc} \frac{2\times 0.386\times 10^{-3}}{C} & \leq & 3.24\\ C & \geq & \frac{2\times 0.386\times 10^{-3}}{3.24}=238.27\;\mu F\;. \end{array}$$

In constant-current mode, the calculation is carried out a little differently. During an 8 ms transmission, the amount of charge consumed from the reservoir capacitor is 28.38mA×8ms=0.227mC. Using the Q=CV capacitor equation, we find that

(9) $$\begin{array}{ccc} Q_{\mathrm{end}} & = & Q_{\mathrm{start}}-0.227\;\mathrm{mC}\\ CV_{\mathrm{end}} & = & CV_{\mathrm{start}}-0.227\;\mathrm{mC} \end{array}$$

(10) $$\begin{array}{ccc} \; & = & C\times 3-0.227\;\mathrm{mC}\;. \end{array}$$

We can again solve for the limit on *C*,

(11) $$\begin{array}{ccc} V_{\mathrm{end}} & = & 3-\frac{0.227\;\mathrm{mC}}{C}\\ & \geq & 2.4 \end{array}$$

or

(12) $$\begin{array}{ccc} C & \geq & \frac{0.227}{3-2.4}=378\;\mu F\;. \end{array}$$

#### 2.2.3. Safety Margins

We remind the reader that the values suggested by these techniques are based on many assumptions, both on the load (e.g., that its power consumption during an activity period is constant) and on the resistor and capacitor. The actual values selected should include safety margins to account for deviation of the actual capacitance from the nominal one (typically 10% or 20%), for possible variation in capacitance due to voltage and temperature, and so on.

### 2.3. Battery-Aware Scheduling

We show below that current limiting does not completely eliminate concentration polarization. That is, even with a current limiter, miniature batteries experience transient reductions in their ability to deliver power. We therefore consider another mechanism to avoid catastrophic failure of the load: skipping activity periods when the supply voltage has not sufficiently recovered to allow the capacitor to safely power the load for another activity period.

The mechanism is implemented in the firmware of the load. The firmware measures the supply voltage before starting the actual power-hungry activity (e.g., generating a radio signal). If it is significantly close to or above the expected starting voltage, the firmware executes the activity period (pulse). Otherwise, one or more activity periods are skipped and the firmware schedules a wakeup to some future time. Upon waking up, the firmware measures the voltage again, and so on, until either voltage recovers and the load can proceed to the actual activity, or until the system fails or enters brownout shutdown due to the voltage dropping too low.

The efficacy of this mechanism depends on the ability of the system to measure its supply voltage and to sleep until a scheduled wakeup, both using very little energy, because during the transient battery failure, these actions are powered mainly by the reservoir capacitor. Our focus in this paper is not on the implementation of this mechanism and in evaluating its energy requirements, but on demonstrating that it is effective under the assumption that it can be implemented in an energy-efficient manner.

## 3. Results

We now present characterizations of the behaviors of miniature batteries carried out using the circuit simulator described in Section 2.2 and using the characterization system that we described in Section 2.1.

### 3.1. Simulation Results

The simulation model shown in Figure 3 can be used to verify the outcomes of a particular set of parameters (limiting resistance, reservoir capacitance, current or power consumption, and so on), as shown in Figure 4, but it can also be used for parameter studies.

**Figure 5:**
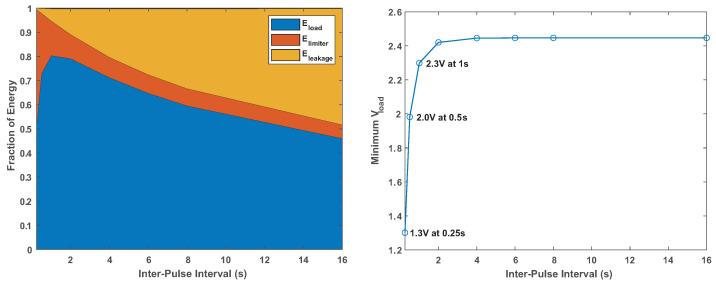
The distribution of battery energy consumption and the minimal voltage seen by the load in simulations of the system with different inter-pulse intervals. The other parameters are the same as in Figure 4.

Figure 5 shows what fraction of the energy drawn from the battery is spent on the load and what fractions are wasted on the current-limiting resistor and on leakage, as well as the minimal voltage seen by the load. The voltage levels remain above 1.8 V down to an inter-pulse interval of 1 or even 0.5 s. The fraction of energy wasted is minimized at inter-pulse intervals of 1 s to 2 s, where is it around 20%. At shorter intervals, a significant fraction of the energy is wasted in the current-limiting capacitor. At long intervals, the energy wasted on leakage becomes significant, reaching almost 50% at 16 s. The energy spent on the limiter drops a little at long intervals, but not significantly.

**Figure 6:**
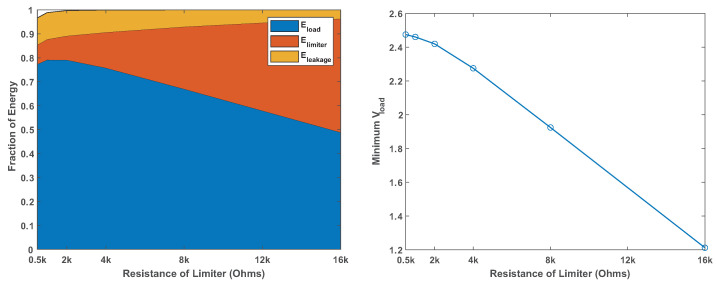
The same quantities plotted in Figure 5, but as a function of the current-limiting resistor. The inter-pulse interval was 2 s in all cases. At the low-resistance end, the fractions do not sum to 1, most likely due to numerical errors in the circuit simulator.

Figure 6 presents a series of simulations designed to help select a current-limiting resistor for a load that consumes 20 mA for 8 ms every 2 s. The graphs show that the best efficiency, around 80%, is achieved with resistors in the 1 k to 2 k range. Even a 4 k resistor, which protects the battery even better, is plausible; efficiency is 76% and the minimal voltage is 2.3 V. At 8 k, the minimal voltage is 1.9 V, too close to the 1.8 V threshold, and efficiency drops further to 67%.

The current-limiting resistor should be chosen using similar simulations. The maximum resistor value should be chosen so as to ensure that the voltage on the load does not drop too low. Below this maximum value, two concerns should drive the choice: the energy wasted in the limiter (favoring smaller values) and the desire to reduce the risk of concentration polarization (favoring larger values).

### 3.2. Experiments Using the Battery-Characterization System: Basic Behaviors

We now present the results of experiments carried out with the battery-characterization system. We start with basic characterizations under constant-current loads of 19 mA for 8 ms every 8 s.

**Figure 7:**
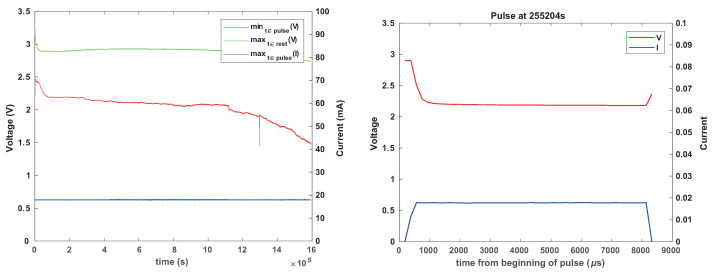
Behavior of Renata CR1625 battery under a constant-current 19 mA load for 8 ms every 8 s. The graph on the left shows the maximum voltage outside activity periods (green) and the minimum voltage and maximum current drawn during activity periods (red and blue). The apparent failure around the second 1,250,000 was caused by a bad connection, not intrinsic battery behavior. The graph on the right shows the behavior during one pulse: while the current is drawn, the battery’s internal resistance causes the voltage to drop, here by about 650 mV, indicating an internal resistance of about 34 Ohms.

Figure 7 shows the behavior of a CR1625 battery under this load. The battery manages to power the load while staying above 1.8 V for at least 1.2 Ms (1.2 million seconds, or almost two weeks), but its internal resistance is significant for this load. Voltage sags during activity periods by about 0.65 V, and the sagging gets worse and worse as the battery drains (internal resistance increases). We conclude that this battery can power this load, but its voltage under load will sag below 1.8 V when it still stores considerable energy.

**Figure 8:**
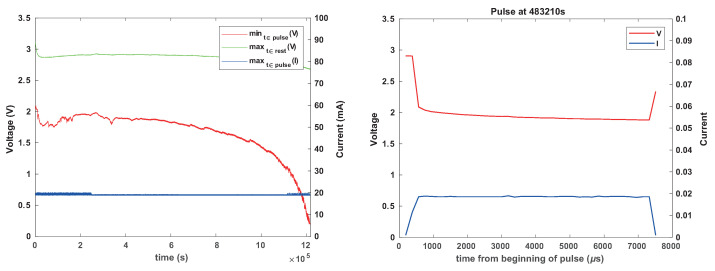
Behavior of a Renata CR1225 battery under the same load. The internal resistance fluctuates; at best, the voltage under load is 2 V (internal resistance of about 47 Ohms), but it is lower than with a CR1625 and it dips close to or below 1.8 V even when the battery is nearly full.

Figure 8 shows that a smaller battery, size CR1225, cannot power this load reliably, at least not without a reservoir capacitor. The graphs show that the internal resistance (voltage sag) is higher and that voltage sags near or below 1.8 V even when the battery is still nearly full.

### 3.3. Using Tantalum and Ceramic Reservoir Capacitors

We now present the behaviors of batteries connected in parallel with a reservoir capacitor. We tested two capacitor models, a miniature 330 μF tantalum capacitor, AVX F950J337KBAAQ2 AVX [21], and a 330 μF ceramic capacitor with an X5R temperature rating ($-55^{\circ }$ to $85^{\circ }$, ±15% capacitance variation over the temperature range), Taiyo Yuden JMK325ABJ337MM-P Tai [22], both rated for 6.3 V. The tantalum capacitor is 3.5 by 2.8 by 1.8 mm and has maximum equivalent series resistance (ESR) of 0.6 Ohms (at 100 kHz). The ceramic capacitor is 3.2 by 2.5 by 2.5 mm, has an effective capacitance of only 220 μF from DC to about 100 kHz, a strong reduction in capacitance at high DC bias (about 40% at 3 V), and extremely low ESR, 1 mOhm at 100 kHz and about 10 mOhm at 1 kHz.

**Figure 9:**
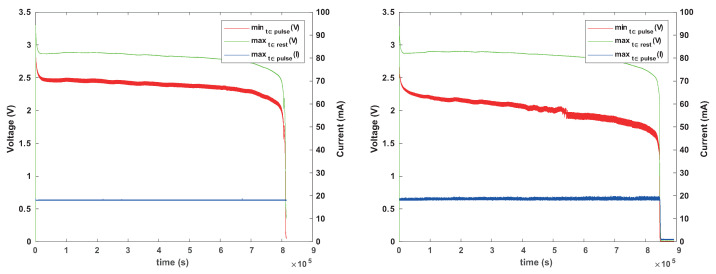
Behavior of Renata CR1025 batteries connected in parallel with a capacitor under pulsed load. The load simulator was programmed to draw 20 mA for 8 ms every second. In the graph on the left, the capacitor was a 330 μF tantalum capacitor; in the graph on the right, it was a 330 μF (nominal) ceramic capacitor with an effective capacitance around 220 μF.

Figure 9 shown the normal behavior of CR1025 batteries connected in parallel with the two types of capacitors (one at a time) under this load. The load was more challenging to support than the load used in the previous experiments: we now programmed the simulator to draw 20 mA for 8 ms every second (as opposed to every 8 s). The ceramic capacitor causes the voltage to drop lower during activity periods than the tantalum capacitor, even initially, because its effective capacitance is lower. The graphs also show two additional behaviors that we do not fully understand, but are not critical. The first is the apparent gradual decrease in the capacitance of the ceramic capacitor, causing the voltage under load to sag lower and lower. The other is variability in the lowest voltage under load.

The main conclusion from these experiments is that an appropriately-sized tantalum capacitor can allow a battery that cannot power the load on its own (a CR1025) to reliably power a load until it is essentially completely depleted. The ceramic capacitor exhibited more erratic behavior and is not recommended. However, further experiments described below indicate that even with such a capacitor, the battery can still fail.

**Figure 10:**
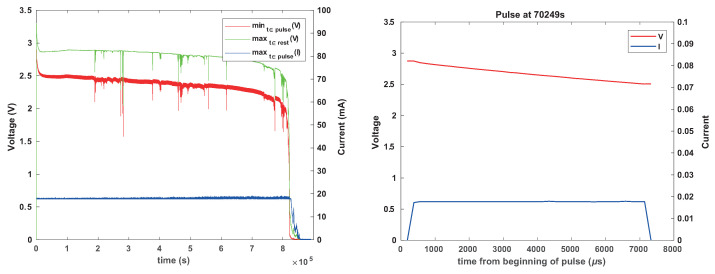
Behavior of another Renata CR1025 battery in parallel with a tantalum capacitor, showing occasional voltage drops, with at least one being catastrophic (below 1.8 V). The graph on the right shows the behavior within one pulse, at a time in which the battery behaved normally. The capacitor and the load were the same as in Figure 9.

**Figure 11:**
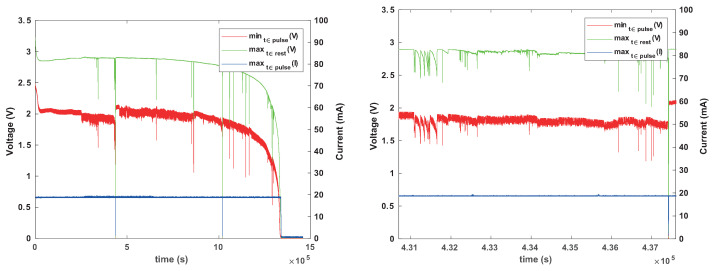
Behavior of a larger Renata CR1225 battery in parallel with a ceramic capacitor, showing occasional voltage drops, with many being catastrophic (below 1.8 V). The graph on the right zooms in, showing interesting patterns of failure after which the battery recovers. The capacitor and the load were the same as in Figure 9.

However, the capacitor can also stress the battery, leading to failure to power the load during an entire activity period. Figure 10 and Figure 11 show such behaviors. In both cases, the battery experiences significant voltage drops, sometimes to well below 1.8 V. The tantalum capacitor mostly induced failures that spanned only one activity period. The ceramic capacitor sometimes induces repeated prolonged periods in which the battery experienced difficulties, perhaps due to its much lower ESR.

These failures appear to be caused by concentration polarization, which can be caused by exposing the battery to a low impedance load, even for short periods.

These failures do not always occur. We repeated the tests with two identical fresh CR1225s, one with each capacitor type; failures were observed only in the battery connected to the ceramic capacitor. We then ran the experiment with two CR1025s connected to two identical tantalum capacitors and two CR1025s connected to two ceramic capacitors. In the second experiment, only one battery failed, the one connected to a tantalum capacitor. We conclude that the propensity for these failures depends on the individual battery; it is not uniform even for batteries from the same batch subjected to the same load and using the same reservoir capacitor.

### 3.4. Experiments with a Real Load

**Figure 12:**
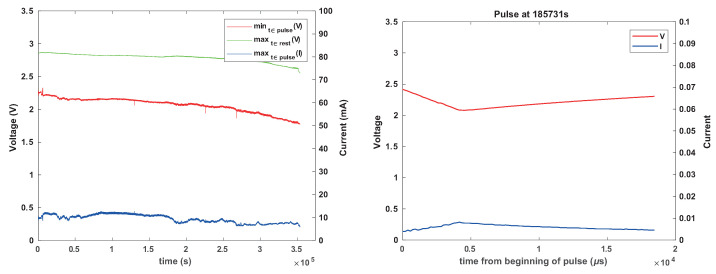
Behavior of a real load (a wildlife tracking tag based on a CC1310 RF microcontroller) powered by a Renata CR1225 battery in parallel with a 330 μF tantalum capacitor. The graph on the left shows the overall experiment and the graph on the right shows the behavior during and after the activity period.

We also demonstrate the real-load mode of the characterization system. Figure 12 shows the behavior of a wildlife tracking tag connected to a CR1225 battery in parallel with a tantalum capacitor. In general, the behavior is similar to that of the simulated load, except that the current consumption is smaller and varies more.

### 3.5. Experiments with a Current-Limiting Resistor

**Figure 13:**
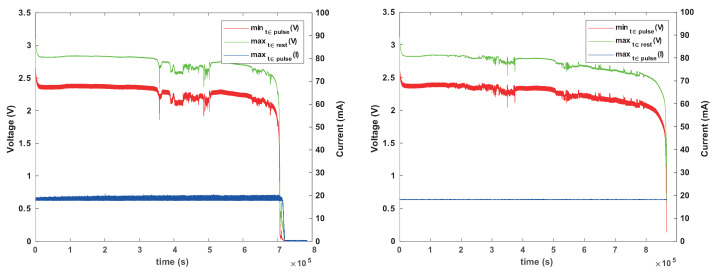
The results of a pulsed current-consumption experiment (20 mA for 8 ms every 1 s) when CR1025 batteries were connected to a 330 μF tantalum reservoir capacitor through a 1 k resistor.

**Figure 14:**
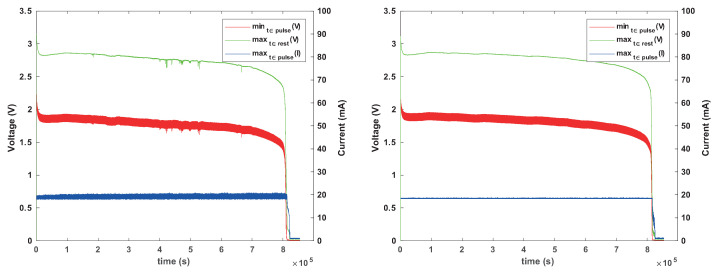
The results of a pulsed current-consumption experiment (20 mA for 8 ms every 1 s) when CR1025 batteries were connected to a 330 μF ceramic reservoir capacitor through a 1 k resistor.

To explore the effectiveness of the current-limiting resistor in mitigating concentration–polarization failures, we ran four experiments with Renata CR1025 batteries from the same batch, all connected to a reservoir capacitor through a 1 k resistor. Two were connected to ceramic capacitors (330 μF nominal, about 220 μF effective) and two to 330 μF tantalum capacitors. The results are shown in Figure 13 and Figure 14. The experiments with tantalum capacitors revealed some transient voltage drops, but much less than the experiment without a resistor (Figure 10). There was also a significant difference in the lifespans of the two batteries, perhaps as a result of these transient failures. The experiments with ceramic capacitors showed no significant voltage drops. The only relevant difference between the two setups is the effective capacitance and we are not sure what caused the difference in behaviors.

To determine whether a larger resistor and larger inter-pulse periods would prevent the transient failures with tantalum capacitors, we repeated the experiment but with two 3.3 k resistors and with 4 s inter-pulse intervals. We observed significant transient voltage drops in some of the experiments, suggesting that a larger current-limiting resistor, at least at these resistances, reduces concentration polarization but does not eliminate it completely. This suggests that a mechanism that allows a load to survive a transient concentration polarization event is indeed required.

### 3.6. Battery-Aware Scheduling

To assess the effectiveness of skipping activity periods to allow the load to survive a concentration polarization event, we ran eight additional experiments that tested this mechanism using an enhanced version of the load-simulation system, in which the firmware implemented this skipping mechanism and was able to report when activity periods were actually skipped. All the experiments used CR1025 batteries and 330 μF tantalum capacitors. Two experiments used 3.3 k current-limiting resistors and inter-activity intervals of 4 s; four experiments used 1k resistors and 1 s intervals; two used no resistor and 1 s intervals. The firmware skipped activity periods when the capacitor and load voltage were less than 2.3 V.

**Figure 15:**
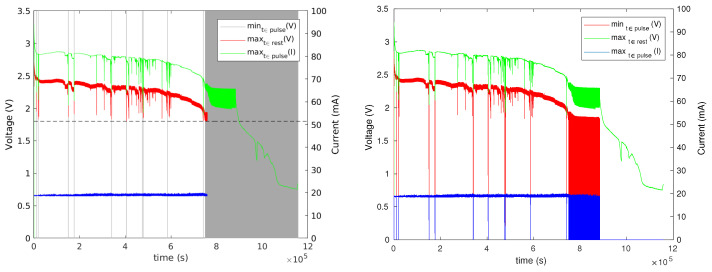
The results of an experiment with a CR1025 battery, tantalum reservoir capacitor, no current-limiting resistor, and with activity-period skipping when the voltage at the beginning of a period is less than 2.3 V. The graph on the left shows measured voltages and currents. The graph on the right shows the same data, but with skipped periods represented by 0 V and 0 A, to show when activity periods were skipped.

**Figure 16:**
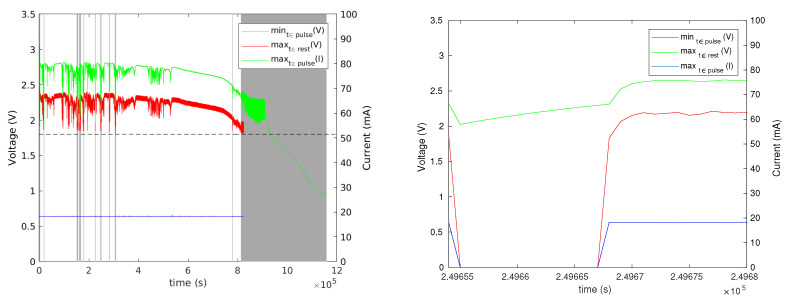
The results of an experiment similar to that shown in Figure 15, but with a 1 k current-limiting resistor. The graph on the right zooms in on one contiguous set of skipped activity periods.

Six of the experiments showed no transient voltage drops below 2.3 V. Two showed such voltage drops, multiple times in both experiments. One of these used a 1 k resistor and the other no resistor. These experiments are presented in Figure 15 and Figure 16. The results indicate that the mechanism is effective; the supply voltage did not drop below 1.8 V even though the batteries experienced voltage drops.

**Figure 17:**
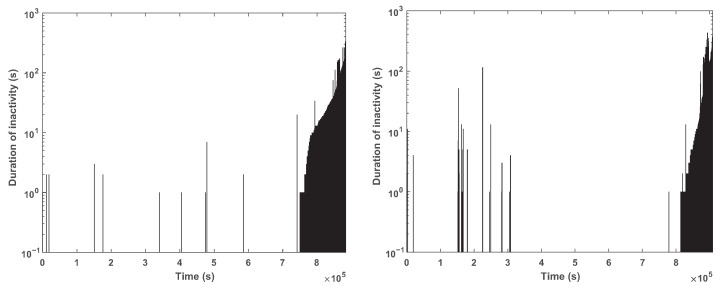
Times at which the load simulator skipped pulses and the duration of the inactivity periods that was required for the battery voltage to rise back above the threshold. The graph on the left shows the result of an experiment with no current-limiting resistor and the graph on the right shows the results with a 1 k resistor.

Figure 17 shows the length of these inactivity periods that the firmware imposes to let the battery recover. Towards the end of life of the battery, these periods become very frequent and longer; an actual load would be observed to “stutter” during this period. In one experiment, the periods of inactivity were short as long as the battery was relatively full, up to about 10 s, with most suspension periods being shorter. In the other experiment, there were several suspension periods that lasted 10 s or more, and one lasted around 100 s.

## 4. Discussion

We have demonstrated that small lithium coin cells that are exposed to periodic low- and medium-impedance loads suffer from transients of high internal resistance. This appears to be caused by a phenomenon called concentration polarization. This happens when the battery powers the electronics directly (consuming about 50 mW for 8 ms every few seconds), when a tantalum or ceramic reservoir capacitor is included in the system, and even when the load and the reservoir capacitor are connected to the battery via a current-limiting resistor. The inclusion of a current-limiting resistor appears to mitigate the effect but not to eliminate it completely.

These observations were made using a low-cost easy-to-replicate system for characterizing miniature batteries operating under pulsed loads. The system is designed to support long-running experiments (days to weeks or more) that are automatically suspended if the computer running the experiment powers down or fails. The low cost and ease of construction of this system allows for concurrent testing of multiple batteries, to obtain statistically valid characterizations at reasonable time scales (that is, they allow for testing multiple batteries concurrently, rather than sequentially).

We also demonstrated that suspending the pulsed load if the battery fails to adequately charge the reservoir capacitor usually allows the battery to recover from concentration polarization before the voltage drops low enough to cause the load to fail or to enter a brownout state (1.8 V in our examples). A firmware mechanism based on this technique has already been implemented in one typical load [6].

**Figure 18:**
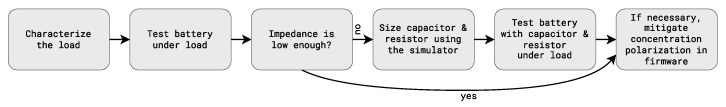
A summary of the characterization and design methodology proposed in this article.

Put together, our results lead to the methodology shown in Figure 18. It begins with a characterization of the load using our system in real-load mode (or an equivalent setup). This characterization should also specify how the load behaves under different supply voltages (e.g., constant current sink, constant power sink, etc.). Then, the battery should be characterized under this load by our system, normally in simulation mode. If the voltage under load remains high enough for most of the life span of the battery, it can be used without a reservoir capacitor and the complexities that it brings. Otherwise, the capacitor and its current-limiting resistor should be selected. The sizing can start with the values derived from the analytical formulas in Section 2.2.2, but should then be optimized using the simulation model from Section 2.2.1. Now, the capacitor and resistor should be connected to the battery and the ensemble should be characterized again in our system, again normally in simulation mode. If the final simulations reveal signs of concentration polarization, the firmware of the target should sense the voltage on the reservoir capacitor and should skip activity periods while it is too low.

## 5. Conclusions

Miniature wildlife tracking tags are often powered by miniature primary batteries. There is a significant gap between the characteristics of these batteries and the needs of these tags. To bridge this gap, we first need to fully characterize the behaviors of these batteries. The system described in this article facilitates this characterization and has revealed a phenomenon not previously documented in such batteries. Other aspects, like the high impedance of miniature batteries, have been known for years.

Hopefully, this characterization system will help expand the range of battery types that are used in wildlife tracking tags. For example, Zinc–Air batteries, which has superior per-gram energy capacity, have not yet been successfully used in such tags.

The gap can be bridged in several ways. Some, like reservoir capacitors and current-limiting resistors, are simple and well known. We have investigated these mechanisms extensively in this article and have shown that tantalum capacitors are more effective than ceramic ones for this function. Other approaches include firmware detection of transient battery malfunctions, the suspension of energy-hungry operations [6], and the development of specialized batteries whose characteristics better match the tags [23]. In the past, analog tracking tags that can be powered by a single 1.5 V Silver–Oxide battery [24] bridged another aspect of the gap. The RF microcontrollers that modern tags are based on cannot be directly powered by 1.5 V cells, but we expect that the general trend towards lower-voltage digital integrated circuits will soon allow for tags to be powered directly by such batteries.

The gap can also be effectively bridged by the incorporation of ultra-low-power energy-harvesting blocks into RF microcontrollers. Such blocks, which could in principle be similar to current stand-alone integrated circuits like the BQ25504 and BQ25570 from Texas Instruments, would take over the management of the battery, the reservoir capacitor, and the tag’s schedule, thereby enhancing both tag life spans and reliability.

## Data Availability

Data available on request.
